# Systems thinking and structural competence in and for medical education

**Published:** 2017-02-24

**Authors:** Marcel D’Eon

**Affiliations:** University of Saskatchewan

In some of my reading and in conversations and meetings, I have noticed two phenomena: on the one hand, an explicit commitment to teaching systems thinking to our students and residents, and on the other, situations in medical education where systems thinking would have made a substantial contribution. The collection of articles in this issue of the CMEJ similarly demonstrate systems thinking or the need for such. Before describing those articles and their connection to systems thinking, I should explain what I mean and how my understanding has been informed by recent national documents and purposeful academic reading.

FMEC 2010^1^ encourages the teaching of systems thinking in the section titled *Promote Prevention and Public Health* (“a multifaceted approach that engages the full continuum of health and health care”) and in the section titled *Medical Leadership* (“Faculties of Medicine must foster medical leadership in faculty and students, including how to manage, navigate, and help transform medical practice and the health care system in collaboration with others”). The system of interest here is clearly health care.

The CanMEDS 2015^2^ Leader role speaks to the engagement of all physicians in improving the health care system, while the Advocate role deals with the determinants of health (most of which are beyond the health care system) and encourages engagement with this broader system (or supporting others who do), “both within and outside of their work environments.” While more encompassing than FMEC 2010, the CanMEDS 2015 framework seems predominantly and heavily focused on work within the health care system.

The concept of structural competence may help overcome the gravitational pull of the health care system to launch physicians and trainees into far flung parts of the social system. Structural competency places emphasis on institutional-level or *structural* interventions: clinicians working collaboratively with community agencies, even non-health sector institutions, and policy makers to affect community and population-level health outcomes.^3^ This is systems thinking boosted with nitrogen tetroxide and hydrazine!^4^

This consistent call for more systems thinking betrays a need, a weakness. For example, some preliminary data indicate that students, during one of their pre-clerkship terms in my medical school, devoted about 10% of their academic time and energy to public health and preventive medicine. This was more than we had anticipated but still much less than the 20% (±5%) proposed in the Medical Council of Canada test blueprint.^5^ These data speak to a lack of attention paid to the health care system itself. Those of us in medical education have an opportunity to consider structural interventions to improve our own system, the curricula we design and deliver.

At a recent meeting where I discussed with other conscientious medical educators and administrators the issue of students turning in assignments late, we decided on a strategy that involved negative sanctions (deduction of marks and meeting the faculty to talk about professionalism). We did not address the systemic causes of the perceived issue, but instead laid blame on the students. At that same meeting, we also discussed the issue of our clinician teachers turning in their exam questions late. We did not suggest that our teachers were unprofessional, but instead we thought of some system specific solutions. Quite a contrast from the way we dealt with the student issue.

It seems that we humans are motivated to assign causes to our behavior and that of others. Attribution is that process by which we explain these causes of behavior, one that is fraught with errors and biases.^6^ From these tenuous assumptions of causes of behaviour, we then create solutions or, in the case of medical schools, policies.

The fundamental attribution^6^ error describes our tendency, in certain situations, to overemphasize personality-based explanations for behavior while underemphasizing situational explanations. The fundamental attribution error flares up mostly when we try to explain the behavior of others. Using the example of late assignments, we presumed students to be poor and unprofessional planners, with unsavory dispositions or attitudes. On the other hand, when evaluating our own behavior, situational factors are often overvalued and exaggerated when there is a negative outcome, while personality and character factors are exaggerated when there is a positive outcome. “I was late because of the train blocking the roadway,” rather than “I managed time poorly and did not leave sufficient time for travel.” “I deserve that award,” even though a cast of hundreds and a few lucky breaks played a huge role in my success! In the example of late exam questions given above, we did not accuse our clinician teachers of unprofessional behaviour, but instead attributed their tardiness to elements of the situation. The fundamental attribution error is alive and well. Systems thinking is often lacking, limited in scope, and laborious to practice. We are fortunate to have many examples of systems thinking and structural competence in this issue of the CMEJ.

Spicer et al., in “Survey evaluation of University of British Columbia residents’ education and attitudes regarding palliative care and physician assisted death,” raise some important and topical issues regarding resident education. Almost two years ago (February 2015), the Supreme Court of Canada struck down the ban on medical assistance in dying (MAiD); the federal government then introduced and passed Bill C-14 to provide a legislative framework for MAiD. Finally, the provincial colleges of physicians and surgeons and health care institutions have been working out the regulations for MAiD at the local levels. Though there has been some research on the attitudes of practicing physicians (and there will be much more), Spicer et al. point out that little research has been done on resident physicians’ opinions on the subject. They conducted a cross sectional anonymous online survey with the resident physicians of British Columbia. From 299 responses, they learned that only 44% of respondents received five or more hours of education in palliative care and 16% received none at all. Shockingly, a full 75% of all respondents had received no education about MAiD whatsoever, while, unsurprisingly, the majority agreed that there should be more education about both palliative care and MAiD. Seen through the lens of systems thinking, we notice not only the obvious gap in training, but especially the juxtaposition of training for both palliative care and MAiD - two aspects of medicine that you won’t likely find as happy bedfellows. Furthermore, despite lack of education and training from their programs, about one-third of residents feel comfortable discussing MAiD with their patients and two-thirds would consider providing MAiD to their patients with the understanding that there would be sufficient safeguards. Perhaps the residents are willing to learn on their own and won’t wait for their residency programs to catch-up.

Martin and her team, in “Exploring the experiences of residents during the first six months of family medicine residency training,” reaffirm that during the shift from undergraduate to postgraduate training residents encounter the reality of practice: the terror of being a real doctor. Martin et al. used interpretative inquiry and monthly, individual, in-depth interviews to explore the residents’ experiences transitioning in a Family Medicine program. They found that through a process of adjustment to substantial increases in responsibility, residents learned what it really meant to be, and become, family physicians. The authors demonstrated a systems thinking perspective when they did not blame the residents for not knowing immediately what to everyone seemed obvious – what a family doctor is - but suggested ways that the program - the system - could adjust to better help the residents to develop an appropriate professional identity.

In “Moral distress and burnout in internal medicine residents” by Sajjadi et al., we are reminded that residents frequently encounter situations in their workplace which may induce moral distress or burnout. The authors measured overall and rotation-specific moral distress and burnout in medical residents at the University of British Columbia, and the relationship between demographics and moral distress and burnout. Forty-five of 88 residents completed the surveys and reported a median moral distress score of 77: quite distressing. In addition, 26% of residents had considered quitting, which they attributed to moral distress, while 21% and 5% had high and low levels of burnout, respectively. This study inclines to systems thinking d avoids the attribution error trap^5^ by comparing different rotations, the environment and the context of medical education – the system again – and does not blame the residents for being weak and ill prepared.

McLeod and Sonnenberg, in “The emotional intelligence of pediatric residents – a descriptive cross-sectional study,” write that many of the social competencies that compose Emotional Intelligence (EI) may have a direct impact on patient care. Using the EQi-2.0© psychometric instrument with 35 pediatric residents at the University of Alberta, Canada, they attempted to describe the EI of pediatric residents. Their overall EI score was not much different from a normative group of college-educated professionals but they had specific strengths (Emotional Expression, Interpersonal Relationships, Empathy, and Impulse Control) and weaknesses (Stress Tolerance, Assertiveness, Independence, and Problem Solving). A systems approach might include linking these findings to “Moral distress and burnout in internal medicine residents” by Sajjadi et al. (also in this issue of the CMEJ) together with other studies^7^ about resident stress in order to uncover a sad, but recurring theme.

In “An examination of Canadian psychiatry residency programs for international medical graduates (IMGs),” Soma et al. identified the relative importance that Canadian program directors of psychiatry place on 43 selection criteria when matching IMGs into their residency programs. They found that academic and behavioral issues of concern were the most important selection criteria, similar, in fact, to what program directors look for most in Canadian graduates. That was a relief! With respect to issues of professionalism, the authors critiqued the Papadakis et al.^9^ article that has misled so many of us to believe that minor infractions in medical school can predict serious unprofessional behaviours out in practice.

In “A digital peer-to-peer learning platform for clinical skills development,” Basnak and the other authors state that medical school curricula may not provide adequate opportunities for pre-clerkship students to practice clinical skills. To address this, medical students developed a peer-to-peer clinical learning program that included student-led objective structured clinical exams (OSCEs). To be clearer and brutally honest, the students’ need provides both evidence for the inadequacy of the instruction in clinical skills and motivation to fill the gap! One hundred and forty-four first-year medical students participated; students wrote case scenarios and then some acted as patients, physicians (the ones being assessed), and evaluators. They put a lot of time and effort into this activity especially for over-busy medical students. Fully 75% of the students said they needed opportunities to practice patient histories and physical exams and that opportunities provided in their medical school curriculum were not sufficient. On the surface, we should at least congratulate the students for their initiative and industry. On a systems level, we might wonder why medical schools do not provide enough instructional time for students to learn these important clinical skills. What other competing areas of study could possibly be taking priority? (To help you arrive at the correct answer to that rhetorical question, I refer you to the editorial from the volume 7(2), “No one is talking about the elephant in the room.”^10^ Please read right to the final few sentences.)

In “IMAGINE-ing interprofessional education: program evaluation of a novel inner city health educational experience,” Hu and her team evaluated student-run interprofessional inner city health delivery and educational program using pre- and post-program surveys. Twenty-eight out of 35 responding participants showed increased understanding of, and comfort with, issues facing underserved populations and resources for underserved populations. Students valued program elements of workshops, shadowing, and the focus on marginalized populations. As with the Basnak et al. paper (above), we might also wonder why we leave the task of organizing, funding and running such successful initiatives to students.

Walsh and her team note in “Residents’ perceptions of simulation as a clinical learning approach” that while simulation is increasingly being integrated into the educational regimen of students and especially residents, there is little research into their perceptions of this learning modality. Learners’ *a priori* perceptions may limit the focus and effectiveness of their learning experiences. The authors conducted 36 semi-structured, one-hour interviews at three time points with 12 residents enrolled in an introductory simulation-based course. Residents believe simulation serves pragmatic purposes, is a safe space to make mistakes, and presents both perils and pitfalls. These authors are systems thinkers as well, evidenced by their recommendation that faculty account for the perceptions of residents to ensure the educational value of simulation is maximized.

“Resetting the compass: exploring the implicit messages of orientation to a community-engaged medical school” uses an apt metaphor in the context of the vast, largely uninhabited region of endless, sometimes frozen, and even beautiful, rock and forest we call northern Ontario. Ellaway et al. explored the implicit and hidden messages within the orientation to the Northern Ontario School of Medicine (NOSM). They used participant surveys, focus groups, and interviews to collect their data which were then analyzed for underlying themes. They found that NOSM’s Orientation Week was generally perceived as a positive and necessary activity. Unsurprisingly, however, there were points of contention and confusion that were unexpected. They found a “hidden curriculum.” These authors showed humility and systems thinking by identifying the hidden curriculum and the unintended perhaps negative consequences of well-meaning policies and activities. For more on the hidden curriculum of orientation, please read “Entitlement and me: problems in Canadian medical education” by Lester Liao^11^ and “Entitlement in medical education: an ongoing discourse” by Sylvia and Richard Cruess and Yvonne Steinert.^12^

Kwok et al, in “Examining the impact of early longitudinal patient exposure on medical students’ career choices,” evaluated the impact of the First Patient Program (FPP) at their school. They thought that most experiences designed to help students make informed career decisions are short and lack long-term encounters with patients. Medical students who completed at least 6-months in the FPP were invited to participate in a survey. A thematic analysis was conducted of their responses. One hundred and forty-eight students participated in the survey. Only 28 (19%) students stated that the FPP informed their career decisions. The authors found that students within the FPP focused mainly on the patient encounter and sought career experiences elsewhere. While the program was popular and seemed to the authors that it would have helped students with their career decisions, the data indicated otherwise. Researchers are sometimes surprised by what they find (as I have been many times over).

Colmers-Gray and team systematically reviewed the published literature on types and frequency of emergency medicine (EM) resident assessments. Reporting of assessment-related costs was a secondary outcome. Seventy-three articles met inclusion criteria. Assessment tools (n=111) fell into 12 categories: mostly simulation-based assessments (28.8%), written exams (28.8%), and direct observation (26.0%). Median assessment frequency (n=39 studies) was only twice per month/rotation. Sadly, no studies thoroughly reported costs. The authors emphatically recommend including cost estimates of assessment programs - important information for system thinkers.

Finally, Litalien, a medical student, exhibits plenty of systems thinking. She laments a lack of engagement on the part of her medical school classmates to debate important issues. She asserts that medical students ought to embrace debate about “the big things—what kind of values we should embrace as medical students, how we can make our classroom environment more inclusive, and whether some of our practices contribute to social inequality.” She is certainly showing leadership and systems thinking when she writes that we all should “engage in debate about our behaviors and, if appropriate, to adjust our practices”.

Besides the valuable content that all these articles bring to medical education, they also show examples of systems thinking and structural competence that unfortunately are too often lacking in our day-to-day decision-making.
